# Epigenetic control of plant senescence and cell death and its application in crop improvement

**DOI:** 10.3389/fpls.2023.1258487

**Published:** 2023-10-30

**Authors:** Yu Zhang, Dongmei Huang, Ying Miao

**Affiliations:** ^1^ Fujian Provincial Key Laboratory of Plant Functional Biology, College of Life Sciences, Fujian Agriculture and Forestry University, Fuzhou, China; ^2^ Department of Biochemistry and Molecular Biology, Xiamen Medical College, Xiamen, China

**Keywords:** cell senescence of plant, DNA methylation, histone modifications, chromatin remodeling, non-coding RNAs, RNA methylation, epigenetic manipulation, crop improvement

## Abstract

Plant senescence is the last stage of plant development and a type of programmed cell death, occurring at a predictable time and cell. It involves the functional conversion from nutrient assimilation to nutrient remobilization, which substantially impacts plant architecture and plant biomass, crop quality, and horticultural ornamental traits. In past two decades, DNA damage was believed to be a main reason for cell senescence. Increasing evidence suggests that the alteration of epigenetic information is a contributing factor to cell senescence in organisms. In this review, we summarize the current research progresses of epigenetic and epitranscriptional mechanism involved in cell senescence of plant, at the regulatory level of DNA methylation, histone methylation and acetylation, chromatin remodeling, non-coding RNAs and RNA methylation. Furthermore, we discuss their molecular genetic manipulation and potential application in agriculture for crop improvement. Finally we point out the prospects of future research topics.

## 1 Introduction

Plant senescence, the last stage of plant development, is a type of programmed cell death at cellular level, occurring at a predictable time and cell (Miao and Zentgraf, 2007). At this stage, there is a functional conversion from nutrient assimilation to nutrient remobilization, which plays a pivotal role in plant architecture and plant biomass, product quality, and horticultural ornamental characters (Guo and Gan, 2005; Lim et al., 2007). Plant senescence can be defined to developmental senescence that is controlled by genetically material affected by inner factors: aging and hormones and stresses-induced senescence that is induced by exogenous environmental stresses: light, water, nutrient, pathogen etc. Even though much progress has been summarized in functional analysis of numerous senescence-associated genes (SAGs) in diverse plant species, especially in *Arabidopsis* leaf senescence, several scientific questions stay unanswered, for examples, what time point plant cell senescence is initiated or how does the action mechanisms of cell senescence of plant is addressed systematically and accurately. As known, cell senescence in plant can occur in predicable space and time during a plant’s lifespan, for example in root cap cell of root, treachery cell of vascular, aerenchym cell and leaf mesophyll cells, petal cell, tapetum cell of anther, macrospore cell, aleuronat layer cell of seed, as well as hypersensitive cell (Gray and Johal, 1998). Increasing evidence indicates epigenetic changes as a cause of cell senescence in organisms (Yang et al., 2023). The integrative regulationary mechanism at the chromatin, transcriptional, post-transcriptional, translational, and post-translational level is introduced to modulate the initiation and process of cell senescence of plant (Woo et al., 2019; Guo et al., 2021; Miryeganeh, 2022). Here, we summarize current research on the mode of action of cell senescence of plant at the epigenetic and epitranscriptional level, including DNA methylation, histone methylation and acetylation, chromatin remodeling, non-coding RNA, and RNA methylation regulation. Moreover, we introduced their molecular manipulation of potential application in plant senescence mediating yield and product quality improvement of agriculture. Finally, the prospects of future research topics were pointed out.

## 2 Epigenetic modifications and their regulation

Epigenetics defines as heritable alterations in chromatin modification and gene expression without any changes in DNA code. In eukaryotes, DNA exists as an intimated complex with histones, which together comprise chromatin polymer. DNA methylation, histone modification, chromatin remodeling, RNA methylation, and non-coding RNA regulation provide a set of interrelated pathways to alter the chromatin conformation to stabilize gene expression in a broad sense. DNA methylation appears in CG, CHG, and CHH contexts (H=A, T, or C), where CG methylation is located in the gene promoter and the body, while non-CG methylation is at TEs for regulating gene expression, ensuring transposon silencing and maintaining genome stability (Law and Jacobsen, 2010). Moreover, the residues of histones tails are subject to covalent modification such as acetylation, methylation, ubiquitination, and phosphorylation under the action of related enzymes. Methylation and acetylation of histones, notably H3 and H4, have been widely studied in yeast, mouse, human and plant. They are thought to correlate positively or negatively with the changes of transcription activity. Adding and removing of chemical group from DNA cytosine or histone residue can alter chromatin structures, thereby affecting transcriptional regulation during the period of plant development and response to a variety of environmental stresses including that of developmental senescence and stresses-induced senescence (Strahl and Allis, 2000). Through the dissociation or reassembly of nucleosomes using energy from ATP hydrolysis is another important mechanism to change the structure of chromatin with loosed or agglutinated status, which mediated by ATP-dependent chromatin remodeling complexes, so that the transcription of specific genes such as senescence associated genes (SAGs) can be selectively “turned on” or “off” (Becker and Horz, 2002). In addition, a huge portion of the genome of eukaryotes is transcribed into non-coding RNAs, which are not translated into proteins but are important triggers for inducing silent chromatin. Currently, increasing evidences reveal that specific mRNA nucleotides can be chemically modified such as m6A and m5C, which can enhance or reduce the binding activity to mRNA of different regulatory factors such as transcription factors, splicing factors, and related enzymes, etc. (Gabriel et al., 2019; Kumar and Mohapatra, 2021; Shen et al., 2023). These epigenetic modifications potentially affect gene transcription, transposon silencing and genomic stability, as well as gene expression of senescence-associated proteins.

## 3 DNA methylation declined with plant senescence

DNA methylation at the fifth carbon of cytosine residues represents a conserved epigenetic mark in eukaryotes that has key function in regulating gene transcription, transposon silencing and genome stability (Law and Jacobsen, 2010; Zhang et al., 2018; Lu et al., 2023). In plants, DNA methylation is established through RNA-directed DNA methylation (RdDM) pathway via DOMAINS REARRANGED METHYLTRANSFERASE 2 (DRM2) (Matzke and Mosher, 2014) and is maintained by three pathways related to CG, CHG and CHH depending on the cytosine sequence context. CG and CHG methylation is balanced by METHYLTRANSFERASE 1 (MET1) and CHROMOMETHYLASE3 (CMT3), respectively, while CHH methylation is maintained by CMT2 or DRM2 (Lindroth et al., 2001; Cao and Jacobsen, 2002; Zhang et al., 2018). DNA methylation can be reversibly erased by 5-methylcytosine DNA glycosylases, such as REPRESSOR OF SILENCING 1 (ROS1), DEMETER, and DEMETER-LIKE (DML) proteins (Choi et al., 2002; Gong et al., 2002; Zhang et al., 2018).

### 3.1 Global dynamics of DNA methylation during leaf senescence

Many studies have reported that dynamic changes of DNA methylation occurred during cell senescence of plant. Ogneva et al. (2016) initially demonstrated the dynamic profile of DNA methylation during cell senescence of *Arabidopsis* shoots aging by using a methylation-sensitive DNA fragmentation (MS-AFLP) assay. They showed a global declining in DNA methylation levels during *Arabidopsis* shoots aging, simultaneously accompanying by a downregulation of *CMT3* and *MET1* methyltransferase genes, and an upregulation of demethylase genes including *ROS1*, *DME*, *DML2*, and *DML3* (Ogneva et al., 2016). By using methylated DNA immunoprecipitation (MeDIP-Seq) assay, comparison of global DNA methylation level between young and senescent cotyledons of *Gossypium hirsutum* L. showed that DNA methylation levels at the promoters, regions around CpG islands, as well as transcriptional termination regions were decreased in senescent cotton cotyledons. The decreased DNA methyltransferase activity was mainly linked to secondary metabolite processes from young to senescence tissue (Dou et al., 2017). Later, by using bisulfite-sequencing analysis, Trejo-Arellano et al. (2020) addressed that local DNA methylation decreased in CHH context during dark-induced senescence, with global loosened changes in chromatin structure in the terminal stage of plant life. However, Zhang et al. (2021) presented the profiles of single-base-resolution DNA methylation of *Moso bamboo* leaves covering the extensive process of vegetative growth and transition to flowering. Their findings indicated that CHH methylated level gradually accumulated from vegetative growth to reproductive development, and genes with CG methylation changes were enriched in ‘vegetative to reproductive phase transition of meristem’ GO term. Integrative analysis of DNA methylation data with RNA-seq data revealed that DNA methylation in various regions of promoters, exons and introns might have different regulatory mechanism to control gene expression (Zhang et al., 2021).

### 3.2 DNA methyltransferase and DNA demethylase affect plant senescence

**Figure 1:**
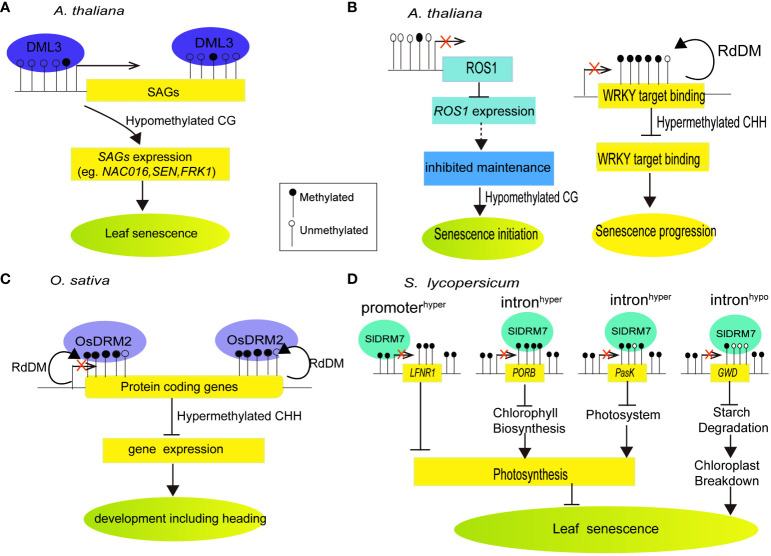
Summarized models of DNA methylation or demethylation by DNA methylases or demethylases regulating target gene transcription and metabolism-related aging in different plant species. **(A)** DNA demethylase DML3 controls leaf senescence in Arabidopsis by reducing CG methylation in hypermethylated gene regions. This activates senescence-associated genes, leading to leaf senescence (Modified from Yuan et al., 2020). **(B)** DNA demethylase ROS1 contributes to leaf senescence in *Arabidopsis*. The decline in *ROS1* expression during senescence indicates reduced demethylation activity, supporting the idea that CG hypomethylation is primarily due to inhibited maintenance rather than active demethylation in the earlier stages. CHH methylation levels in WRKY target bindings are notably elevated during late senescence stages (Modified from Vatov et al., 2022). **(C)** DNA methylase OsDRM2 controls development and heading in rice. OsDRM2 is required for most of the CHH methylation in rice, predominantly in small TEs such as MITEs t located near the end of protein coding genes (Moritoh et al., 2012; Tan et al., 2016). **(D)** Tomato DNA methylase SlDRM7 regulates leaf chlorosis and senescence. Silencing SlDRM7 alters DNA methylation and transcript levels of genes related to chlorophyll synthesis, photosynthesis, and starch degradation, resulting in leaf senescence and chlosis (Modified from Wen et al., 2022).

Several DNA methylase and demethylase enzymes have been studied to provide mechanistic insight into global changes in DNA methylation during cell senescence of plant leaf. In *Arabidopsis*, neither overall nor spatial reduction of DNA methyltransferase MET1 activity by a *met1* mutation or local expressed MET1 antisense gene driven by DEMETER (DME) promoter, led to global DNA hypomethylation and developmental defects including reduced fertility, failed flowering, or greatly delayed plant senescence (Kim et al., 2008). Whole-genome bisulfite sequencing (WGBS) were performed in *Arabidopsis* leaves of *demeter-like 3* (*dml3*) mutant that lost the DEMETER-like DNA demethylase, relative to wild-type at three developmental stages, namely NS (non-senescent), ES (early senescence)), and LS (late senescence). While a reduction of genome-wide methylation levels at CG context was observed at stage transition from NS to LS in wild-type, loss of *DML3* led to significant increase of DNA methylation at the promoters of several senescence-associated genes, thereby suppressing their expression and delaying leaf senescence. These results demonstrated that DML3 activating DNA demethylation and expression of selected senescence-associated genes modulated leaf senescence (Yuan et al., 2020) (**Figure 1A**). More recently, Vatov et al. (2022) reported that initial cytosine methylation at CG context declined moderately during progressing leaf senescence, while moderate *de novo* methylation of cytosines at CHH context associated with late senescence. Moreover, hypermethylated mutant *ros1* and hypomethylated triple mutant *dmr1/2 cmt3* (*ddc*) displayed a faster senescence progression and enhanced nitrogen remobilization from the leaves. Loss of methylation in CHG and CHH contexts at W-box element targeted by WRKYs as well as a core motif recognized by bZIP transcription factors (ACGTG) was observed in *ddc.* Differentially methylated regions at *ROS1* promoter were related to down-regulation of *ROS1* with the progression of senescence (Vatov et al., 2022) (**Figure 1B**). It seems that the DNA methylation during leaf senescence decrease because of cytosine methylation maintenance inhibition. Nonetheless, the linkage between moderate methylome changes and up-regulated genes during leaf senescence remains unclear.

In rice, disruption of *DOMAINS REARRANGED METHYLASE 2* orthologous (*OsDRM2*) displayed abnormal DNA methylation and developmental tissues like delayed or absent heading or growth defects (Moritoh et al., 2012). Mutations in OsDRM2 cause near-complete CHH methylation loss and derepression of small transposable elements such as MITEs. These MITEs are commonly found at the ends of protein-coding genes. In *osdrm2* plant, there is a significant reduction in CHH methylation at the ends of protein-coding genes. This finding suggests that OsDRM2-dependent CHH methylation primarily focuses gene-associated small TEs, which consequently impacts the methylation of protein-coding genes and promotes plant senescence (Tan et al., 2016) (**Figure 1C**). Silencing the elongation complex protein 2-like gene (*SlELP2L*) inhibited leaf growth, accelerated leaf and sepal senescence, and produced dark green fruit. Gene expression analysis revealed that the SlELP2L-deficient plants had up-regulated gene expression level of DNA methyltransferases, implying that SlELP2L may play a role in DNA methylation in tomato (Zhu et al., 2015). Meanwhile, Domains Rearranged Methyltransferase7 (SlDRM7), a regulator of leaf senescence of tomato, was activated by aging- and dark-induced senescence. Silencing of SlDRM7 causes *SlLFNR1’s* promoter to become hypermethylation, *SlPORB’s* and *SlPsaK’s* introns to become hypermethylation, and *SlGWD’s* intron to become hypomethylation, resulting in their expression repression, leads to the inhibition of photosynthesis and starch degradation, which ultimately leads to leaf senescence and chlorosis. Additionally, SlDRM7 can be induced by leaf senescence, creating a feedback regulatory loop that balances vegetative growth and leaf senescence (Wen et al., 2022) (**Figure 1D**). Further, Li et al. (2022) reported that leaf senescence of vegetable pak choi was accelerated by treatment with DNA methylation inhibitor 5-Azacytidine. Several DNA methyltransferases were down-regulated in leaves during pak choi storage. By using bisulfite-sequencing assay, it showed that DNA methylation enrichment in the promoter regions of senescence related genes *BcSGR2* and *BcSAG12* were decreased during storage. These results demonstrated that DNA demethylation was observed in association with the leaf senescence of pak choi.

### 3.3 SAG Loci-specific changes in DNA methylation

It is important to note that DNA methylation significantly affects leaf senescence by altering chromatin structure in several ways. He et al. (2018) initially discovered a naturally occurring methylation region, *NMR19-4*, in *Arabidopsis* that was identified in the promoter region of the *PHEOPHYTIN PHEOPHORBIDE HYDROLASE* (*PPH*) gene, and the methylation status of *NMR19-4* was associated with repressed *PPH* expression and delayed leaf senescence. Epi-allelic variations at key senescence associated genes have also been demonstrated to regulate leaf senescence in several other crop species. In maize, alterations in DNA methylation were observed during critical life event transitions when silencing the Mutator-Don Robertson transposable elements and were proposed to contribute to whole-plant senescence (Li et al., 2010). In barley, a decrease in DNA methylation was observed in senescing leaves at a specific CpG motif located within the promoter region of *HvS40* (Ay et al., 2015). Similarly, tomato fruit ripening was accompanied by changes in the DNA methylome. The active DNA demethylation of promoters of fruit-ripening genes bearing a binding site for RIPENING-INHIBITOR was correlated with the transcriptional inhibition of these genes (Zhong et al., 2013; Lang et al., 2017).

## 4 Histone methylation and acetylation antagonistically regulate plant senescence

Histone modification and histone modifier enzymes play a vital role in regulating gene expression by modifying the chromatin component. Lysine 4 demethylation and trimethylation on histone 3 protein (H3K4me2, H3K4me3) as well as lysine 9 acetylation on histone 3 protein (H3K9ac) are active histone modifications associated with leaf senescence. Two silence marks, lysine 27 dimethylation and trimethylation (H3K27me2, H3K27me3), are also linked to leaf senescence (Brusslan et al., 2012; Brusslan et al., 2015; Zhang et al., 2022).

### 4.1 Genome-wide histone modifications during leaf senescence

The combination of chromatin immunoprecipitation sequencing (ChIP-seq) and RNA-seq methods has been employed to explore the genome-wide landscape of histone modifications and gene transcription during leaf senescence. Brusslan et al. (2015) demonstrated, in *Arabidopsis*, that senescence-associated genes (SAGs) correlates with elevated levels of H3K4me3 and H3K9ac, while the inhibition of senescence-downregulated genes is related to H3K27me3 (Brusslan et al., 2012; Wang et al., 2019). Zhang et al. (2022) further discovered an increase in the levels of genome-wide H3K9 acetylation during age-dependent senescence in the flag leaf of rice. The findings revealed a coordination correlation between the breadth and density of H3K9ac and both gene transcription initiation and transcript elongation. Integrative analysis revealed a landscape of H3K9ac-associated differentially expressed genes, including SAGs, metabolism-related genes, and genes related to miRNA biosynthesis. These results suggest a complex regulatory network of metabolism-mediated senescence that is associated with H3K9ac during rice flag leaf senescence.

### 4.2 Histone methyltransferase and histone demethylase affect leaf senescence

Ay et al. (2009) provided the first evidence linking histone methylation and leaf senescence regulation. Upon activation of *WRKY53*, a key regulator of leaf senescence, levels of H3K4me3 and H3K4me2 were observed significantly increased at the coding regions and 5’ end of *WRKY53*. The overexpression of SUVH2, a histone methyltransferase, however, suppressed the transcriptional initiation of *WRKY53* and additional SAGs, thereby leading to a delay of leaf senescence (Ay et al., 2009). Subsequent research indicated that overexpression of *SUVH2* affected the gene expression regulation of approximately 50% of the 380 senescence-related regulatory factors (Ay et al., 2014). Therefore, SUVH2 has a significant effect on leaf senescence processes. Numerous studies have established several related enzymes controlling histone demethylation and affecting leaf senescence. For example, Liu et al. (2019) discovered that JMJ16, a particular H3K4 demethylase, negatively controlled leaf senescence, mainly by downregulating the expression of positive senescence regulators, *WRKY53* and *SAG201*, by decreasing the H3K4me3 levels at these regions to avoid premature leaf senescence in mature leaves. Wang et al. (2019) reported that H3K27me3 methylation inhibited the transcriptional activation of key *SAGs* in *Arabidopsis*. They discovered that RELATIVE OF EARLY FLOWERING 6 (REF6), a H3K27me3 demethylase, directly accelerated the expression of ten specific *SAGs*, and loss-of-function of REF6 postponed leaf senescence by increasing H3K27me3 levels at all the target *SAGs*. Recently, in tomato plant overexpressing SlJMJ4, a histone H3K27 demethylase, resulted in premature senescence and enhanced dark-and ABA- induced leaf senescence (Ding et al., 2022). Under dark conditions, SlJMJ4- induced leaf senescence was linked to the upregulation of *SlORE1*, *SlNAP2*, *SlSAG113* and *SlSAG12* by removing H3K27me3. When responding to ABA, the overexpression of SlJMJ4 resulted in an increased binding ability to the loci of *SlORE1*, *SlSAG113*, *SlNAP2, SlSAG12, SlNCED*3 and *SlABI5*, while decreasing their H3K27me3 levels (Ding et al., 2022).

### 4.3 Histone acetyltransferase and histone deacetylase affect leaf senescence

Histone acetylation is another extensively investigated histone modification that plays a role in leaf senescence. It is generally linked to gene activation, in contrast to histone deacetylation that is associated with gene silencing. The process of histone acetylation is a reversible and is catalyzed by the enzymes histone acetyltransferases (HATs) and deacetylases (HDACs). The earliest study exploring the relationship of histone deacetylases and leaf senescence is AtHD1 (Tian and Chen, 2001). Transgenic plants with antisense-AtHD1 had reduced *AtHD1* level and increased levels of tetra acetylated histone H4 resulting in early leaf senescence and other developmental abnormalities (Tian and Chen, 2001). AtHDA6, a type of RPD3-like histone deacetylase, has been implicated in *Arabidopsis* leaf senescence, jasmonate response, and flowering. In comparison to the wide type (WT), the Arabidopsis HDA6 mutant exhibited higher levels of acetylated H3 and prolonged leaf lifespan (Wu et al., 2008). AtHDA9, a RPD2-like histone deacetylase, was found to play a pivotal role in promoting the onset of leaf senescence. Loss-of-function *HDA9* delayed leaf senescence and increased H3K27ac levels. HDA9 formed a repressor complex with POWERDRESS and WRKY53 to suppress the expression levels of negative senescence regulators (*WRKY57*, *NPX1*and *APG9*) by removing H3 acetylation marks, thereby promoting aging in *Arabidopsis* (Chen et al., 2016). AtHDA15, another histone deacetylase, was reported that it was recruited by the single-stranded DNA-binding protein WHIRLY1 to target *WRKY53* loci and reduced H3K9ac enrichment of *WRKY53* promoter region, thus repressing its transcription and inhibiting leaf senescence in *Arabidopsis* (Huang et al., 2018; Huang et al., 2022). Further integrative analysis of the genome-wide H3K9 acetylome and transcriptome of *hda15* and *hda15 why1* double mutants relative to WT revealed that HDA15 had deacetylase activity and was able to remove H3K9ac at the targeted promoter region repressing the expression of senescence up-regulators, *LOX2* and *LARP1C* and delaying leaf senescence. Additionally, AtHDA15 can be recruited by WHIRLY1 to the regions near the transcription start site (TSS) of nutrient recycling-related genes (*GSTF10, DTX1*, *ABCC9*), the D1 synthesizer attenuator *PDIL1-2* of photosystem II protein, as well as *WRKY53* and *ELF4*. This recruitment removes H3K9ac from their promoter region leading to the repressing of gene expression and the delaying both leaf senescence and flowering during the early stage of plant development (Huang et al., 2022). In rice, overexpression of *OsHDA710*, which encodes a histone deacetylase, delays leaf senescence, and knockdown *oshda710*accelerates leaf senescence. In particular, the overexpression of *OsHDA710* induces up-regulation of genes related to photosynthesis and chlorophyll biosynthesis, while downregulating certain genes associated with programmed cell death and disease resistance (Zhao et al., 2020). However, up to now, only one histone acetyltransferase, AtHAC1, has been reported in *Arabidopsis* to play a role in promoting leaf senescence and regulating the expression of *ERF022* by H3K9ac enrichment (Hinckley et al., 2019).

### 4.4 SAGs specific loci changes in histone modification

**Figure 2:**
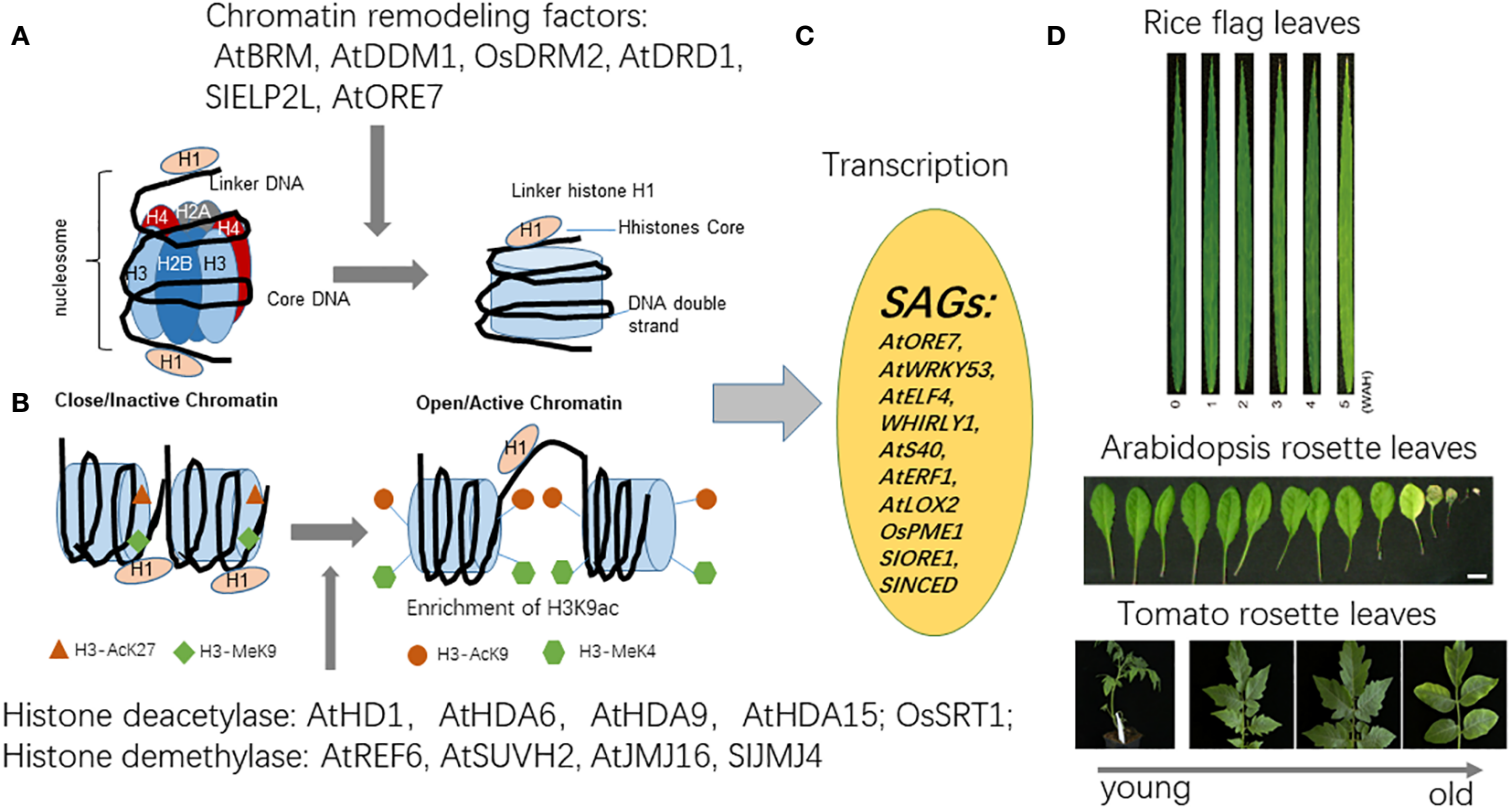
The mechanism of chromatin remodeling and histone modification and related factors regulating plant leaf senescence. Plant leaf senescence is regulated by epigenetic modification including histone methylation, histone acetylation and chromatin remodeling. **(A)** The chromatin decondensation is repressed by DDM1 and DRD1, members of the SWI/SNF family of chromatin remodelers affects *ORE7* transcription and leaf senescence. **(B)** Several histone modification enzymes such as histone acetylases OsHD1, AtHDA6, AtHDA9, AtHDA15 and histone methylases OsSRT1, SlELP21, AtSUVH2 have been reported to regulate leaf senescence in *Arabidopsis* and crop plants. **(C)** Histone modification mediates genetic reprogramming, the regulation of SAGs transcription such as *AtWRKY53*, *AtERF6*, *AtERF1*, *AtELF4* in *Arabidopsis*, *OsRE7* in rice, *HvS40* in barley, is indicated. **(D)**. The images of rice flag leaf aging and leaf senescence in *Arabidopsis* and tomato.

Numerous senescence-associated genes (SAGs) in crop plants were identified in impact of histone modification enzymes. For example, during leaf senescence, the expression of the barley gene *HvS40*, which encodes a potential regulator of leaf senescence, is significantly up-regulated. At the onset of leaf senescence, the regions of promoter and coding sequence of *HvS40* enriched H3K9ac, but declined the enrichment of H3K9me2 (Ay et al., 2015). Under drought conditions, H3K9ac enrichment at the promoter and coding sequence regions of *HvS40* in barley was affected by single strand DNA/RNA binding protein HvWHIRLY1 protein, promoting leaf senescence (Janack et al., 2016). Conversely, AtWRKY53, a leaf senescence marker regulator in *Arabidopsis*, its expression was repressed by AtWHIRLY1 through AtWHIRLY1 recruiting HDA15 at the *TSS* region of *AtWRKY53* promoter to remove H3K9ac, repressing leaf senescence (Huang et al., 2018; Huang et al., 2022). In rice, OsSRT1 is an NAD+-dependent histone deacetylase. The enriched H3K9ac level at the senescence marker gene Os*SAG12* in *OsSRT1* RNAi line was exhibited, resulting in accelerated leaf senescence and programmed cell death (PCD) and aging (Fang et al., 2016). Zhong et al. (2013) further found that OsSRT1 played a crucial role in negatively regulating leaf senescence by repressing gene expression in the MeOH-jasmonates metabolic cascade. Notably, this effect was achieved, in part, through the deacetylation of histone H3K9 in *OsPME1* (Zhong et al., 2013). In tomato, under dark conditions and response to ABA, the H3K27me3 enrichment of a series of senescence marker genes such as *SlORE1, SlNAP2, SlSAG113, SlSAG12, SlNCED3*, and *SlABI5* loci was removed by SIJMJ14, inducing leaf senescence (Ding et al., 2022) (**Figure 2**).

Therefore, active histone modifications such as H3K4me3 and H3K9ac positively associated with SAGs transcription via activating transcription initiation and elongation to accelerate leaf senescence, and silence marks such as H3K27me3 and H3K9me3 negatively linked to SAGs transcription and repress leaf senescence. However till now, only a few histone methyltransferase or acetyltransferase was identified, mechanism of controlling the dynamic of histone modification during cell senescence of plant maintains challenging.

## 5 Chromatin remodeling is involved in plant cell senescence

Chromatin remodeling is a change in the structure of chromatin, which means that the structure of chromatin is loosed or agglutinated through the dissociation or reassembly of nucleosomes mediated by ATP-dependent chromatin remodeling complexes, so that the transcription and expression of specific genes can be selectively “turned on” or “off”. ATP-dependent chromatin remodeling complex is highly conserved across evolution.

Chromatin remodeling complexes consist of several protein families, all of which contain an ATP-hydrolyzing DNA translocase subunit of the sucrose nonfermenting 2 (Snf2). These complexes catalyze changes in nucleosome position, occupancy, or/and composition to regulate DNA and core histones interactions, thereby remodeling chromatin locally or globally to adopt or impede regulatory proteins. On the basis of ATPase/helicase like regions (Snf2 domain) characteristics, chromatin remodeling proteins have been briefly classified into six groups and 24 subfamilies, each with specific functional properties (Flaus et al., 2006; Gentry and Hennig, 2014; Song et al., 2021). Snf2 family genes have been identified in model plants *Arabidopsis*, rice, tomato, barely and soybean with 41, 40, 45, 38, and 66 members, respectively (Knizewski et al., 2008; Hu et al., 2013; Zhang et al., 2019; Chen et al., 2022; Wang et al., 2023). Roles of chromatin remodeling complexes during plant development and stress response have been highlighted in many reviews (Han et al., 2015; Ojolo et al., 2018; Jian et al., 2021; Song et al., 2021; Bieluszewski et al., 2023). This part discusses the advances of chromatin remodeling factors in plant senescence and cell death.

### 5.1 Implications of chromatin remodeling activities in the leaf senescence

Chromatin conformation is dynamically changed during cell senescence induced by age and environmental cues (Fal et al., 2021; Song et al., 2021; Miryeganeh, 2022). Ay et al. showed the global changes in chromatin organization during age-dependent leaf senescence, including the disassembly of chromatin center, the degradation and speckled distribution of euchromatin, the disappearance of nucleolus (Ay et al., 2009). The heterochromatic regions were distinguished from euchromatic regions during leaf senescence with different histone marks, influencing the accessibility of transcription factors to cis-regulatory elements. Chromatin compaction is also supposed to be involved in gene regulation in leaf senescence induced by unsuitable light intensity (van Zanten et al., 2012; Li et al., 2023). A decrease of chromatin compaction in chromocenters was observed in seedlings exposed to shade. Photoreceptor phyB was reported to inhibit dark-induced leaf senescence (Sakuraba et al., 2014) and loss of phyB leads to lower chromatin density comparing with the wild-type under normal light conditions (Tessadori et al., 2009). Another Photoreceptor CRY2, which suppresses leaf senescence in response to blue light, facilitates chromatin decompaction under low light intensity (van Zanten et al., 2010; Kozuka et al., 2023). Thus, dynamic chromatin reorganization is an important mechanism for controlling leaf senescence by altering gene expression patterns (**Figure 2**).

### 5.2 Functions of chromatin remodeling factors during plant senescence

Ectopic overexpression of ORE7/ESC that is a chromatin architecture-controlling protein containing an AT-hook DNA-binding motif extended leaf longevity and post-harvest storage life, suggesting a possibility that alteration of chromatin architecture may be a mechanisms to control plant senescence (Lim et al., 2007). A heterozygous line (*ORE7/ore7-1D)* with lower expression of the activated *ORE7* genes was used in this study to analyze the senescent phenotypes. *ORE7/ore7-1D* mutant showed a delayed senescence phenotype as well as globally altered gene expression. Moreover, more reticular chromatin distribution and intensely nuclear bodies than wild-type plants were observed in *ORE7/ore7-1D* mutant. It is possible that ORE7 binds to AT-rich DNA sequences and modifies the chromatin architecture in the nucleus, then leading globally altered gene expression. In addition, a subset of hormones (jasmonic acid, abscisic acid, ethylene, and salicylic acid) related genes was down-regulated in *ORE7/ore7-1D* mutant, suggesting ORE7 repressed the signaling pathway of hormones during plant senescence (Lim et al., 2007).

Two SWI2/SNF2 chromatin-remodeling proteins, namely, chromatin remodeling protein 1 (DEFECTIVE IN RNA-DIRECTED DNA METHYLATION 1, DRD1) and ATP-dependent DNA helicase DDM1 (DECREASED DNA METHYLATION 1), were identified as regulators of leaf senescence (Cho et al., 2016). *drd1-6* and *ddm1-2* mutants, which both have the occurrence of mutations in the helicase superfamily C-terminal (HELICc) domain, showed delayed leaf senescence during dark-induced senescence and natural senescence. *drd1-6* mutants exhibited higher photosynthetic parameters and lower expression levels of *SAG12* and chlorophyll degradation-related genes than wile-type plants during dark-induced senescence. Moreover, the transcript levels of 180-bp centromeric (*CEN*) repeats and pericentromeric repeats termed transcriptionally silent information (*TSI*) showed slower and lesser increase in the *drd1-6* mutant than in the wile-type plants. Similarly, *ddm1-2* also exhibited a longer stayed green phenotype and higher photochemical efficiency than wild-type plants (Cho et al., 2016). Therefore, the authors concluded that the ATP-helicase domain might be key component of SWI2/SNF2 chromatin remodelers for regulation of leaf senescence.

Several members of the SWI2/SNF2 complex were also reported to regulate the expression of SAGs during plant senescence, but the exact function is still indistinct. BRAHMA (BRM) is one member of the two catalytic ATPase subunits of the SWI2/SNF2 complex. It directly targets to a large amount of SAGs (Archacki et al., 2013; Li et al., 2016). The H3K27me3 demethylase REF6 facilitates the recruitment of BRM and promotes leaf senescence by activating numerous senescence regulatory and functional genes (Li et al., 2016; Wang et al., 2019). Based on the data of ChIP-chip and ChIP-seq, BRM was reported to target to the promoter region of a large number of SAGs directly (Li et al., 2016; Archacki et al., 2017). Moreover, yeast two-hybrid screens showed BRM may interact with numerous leaf senescence regulators (Efroni et al., 2013), such asWRKY53, ABF3 (abscisic acid responsive elements-binding factor 3), CRF6 (cytokinin response factor 6), WRKY6, RSL1 (RHD SIX-LIKE 1), MYC2 (Basic helix-loop-helix (bHLH) DNA-binding family protein), NAC046, NAC083 (NAC domain containing protein), HB40 (homeobox protein 40), TCP1, TCP4, TCP5, TCP16 (TCP family transcription factor), etc. In addition, BRM can interact with UPL3 and UBP12 to maintain BRM polyubiquitination levels, involving in metabolic cell senescence (Lan et al., 2022). Another catalytic ATPase subunits of the SWI2/SNF2 complex, SYD (SPLAYED) plays both redundant and differential roles with BRM during plant development (Bezhani et al., 2007). It was reported to co-target to a number of senescence related genes such as *NAC083*, *NAC032*, *NAC019*, *PIF4*, *WRKY6* etc. with BRM (Shu et al., 2021), suggesting that SYD and BRM may have redundant roles in regulation of leaf senescence. In addition, genome-wide analysis showed that loss of multiple chromatin remodelers upregulated SAGs (Archacki et al., 2017; Shu et al., 2021), while the genes coding chromatin remodelers such as *CHR10*, *ALTERED SEED GERMINATION 3* (*ASG3*) and *CHR19* (*ETL1*) were shown to be upregulated during senescence (Breeze et al., 2011). Further studies are necessary to uncover roles of diverse chromatin remodelers in leaf senescence and to determine how various epigenetic modifications coordinately to respond to different internal and external factors during leaf senescence in the future.

## 6 Non-coding RNAs and plant senescence

Non-coding RNAs can be classified into three categories according to nucleotide numbers of their length: small RNAs (18-30 nt), including miRNAs, siRNAs and piRNAs; medium-sized RNAs (50-300 nt), including snoRNAs and snRNAs; and long non-coding RNAs (lncRNAs) over 200 nt in length (Wang et al., 2017). miRNAs and siRNAs are two primary classes of small RNAs involved in mediating gene expression, while snoRNAs and lncRNAs are represented novel classes epigenetic regulators. SnoRNAs are mainly involved in the biogenesis of ribosomes. With the development of DNA-RNA interaction detection technique, a large number of snoRNAs have been found in *Arabidopsis* and rice to retain chromatin and regulate the structure of chromatin and gene expression through interaction with H3K4me3-labeled gene sites (Chen et al., 2018; Xiao et al., 2022). LncRNAs play critical roles in plant development and environmental responses. Some lncRNAs interact with histone-associated methylases and acetylases to recruit chromatin modification complexes that regulate gene expression at the chromatin level (Zhang et al., 2019).

### 6.1 microRNAs related to cell senescence of plant

microRNAs (miRNAs) are short, non-coding RNAs that are highly conserved and typically range in length from 20 to 24 nucleotides. The discovery of lin-4, the first miRNA, in 1993 (Lee et al., 1993), in nematode *Caenorhabditis elegans*, marked the beginning of miRNA research, and since then, researchers have identified thousands of miRNAs in both plants and animals. These miRNAs play diverse roles in regulating a wide range of biological processes (Ameres and Zamore, 2013; Dexheimer and Cochella, 2020). miRNAs are essential in post-transcriptional gene silencing, where they suppress gene expression by either triggering the cleavage of complementary mRNAs or inhibiting their translation (Bartel, 2009). Although many factors involved in the processes of miRNA biogenesis, conversion, mobilization, and action in plants, miRNAs regulate target genes through transcript cleavage, translational repression, and enhancing transcription (Rogers and Chen, 2013; Xie et al., 2015; Yu et al., 2017). These regulatory mechanisms have been extensively reviewed (Pulido and Laufs, 2010; Woo et al., 2019; Swida-Barteczka and Szweykowska-Kulinska, 2019; Ostrowska-Mazurek et al., 2020; Guo et al., 2021; Miryeganeh, 2022). Only a few miRNAs have been functionally characterized with specific roles in the regulation of plant senescence. An example of miRNA regulation in leaf senescence and programmed cell death is miR164, which selectively targets NAC-domain containing proteins, including NAC2, KIR1, and ORE1 in *Arabidopsis*, *Sorghum*, and *Populus* (Kim et al., 2009; Li et al., 2013; Kim et al., 2014; Qiu et al., 2015; Fujimoto et al., 2018; Gao et al., 2018; Kim et al., 2018; Wang et al., 2021). AtORE1 is known to be post-transcriptionally regulated by miR164 in *Arabidopsis* leaf senescence (Kim et al., 2009); *MIR164* transcription is inhibited by the direct binding of EIN3 to its promoter. The EIN2-EIN3-ORE1/MIR164-ORE1 pathway is the effector of a signaling cascade of ethylene, a hormone known to accelerate senescence (Li et al., 2013). AtKIR1, another identified NAC TF, possesses the recognition site for miR164, which induces programmed cell death in stigma and in shoots (Gao et al., 2018); and the sorghum bicolor orthologous of KIR1 (D) induces programmed cell death in pitch parenchyma of stalks, it might facilitate nutrient remobilization from source to sink tissues (Gao et al., 2018). miR396 limits growth-regulating factors (*GRF*) gene expression. It targets seven out of nine *Arabidopsis* GRFs (Jones-Rhoades and Bartel, 2004). The miR396 level positively correlates with the age of the leaves; it increases while leaf cells proliferate and after the proliferation arrest (Debernardi et al., 2014); miR319 is another miRNA that represses a group of transcription factors known as TEOSINTE BRANCHED/CYCLOIDEA/PCF (TCP). It is responsible for the biosynthesis of jasmonic acid, accelerating vessel formation that linked to the intensification of secondary cell wall biosynthesis and the initiation of programmed cell death, ultimately impacting leaf development and promoting the senescence process (Schommer et al., 2008; Seltmann et al., 2010; Koyama et al., 2017; Sun et al., 2017; Bresso et al., 2018). A recent study addressed the function of miR840–PPR/WHY3 module in *Arabidopsis* leaf senescence. The results demonstrated that the accumulation of short maturation products of miR840 was correlated with the progression of leaf senescence in *Arabidopsis*. Conversely, knockdown of miR840 resulted in delayed plant senescence, whereas overexpression of *MIR840* enhanced senescence symptoms. miR840* and miR840 worked together to target the same pair of genes, *PPR* and *WHY3*, through cleavage transcripts or repressing translation, respectively, reprogramming a series of downstream SAGs (Ren et al., 2022). High-throughput small RNA sequencing techniques have been utilized to identify senescence-inducing miRNAs in *Arabidopsis*, rice, maize, and tomato. These miRNAs regulate several important pathways including hormone signaling, nutrients remobilization, and response to oxidative stresses during plant senescence (Xu et al., 2014; Thatcher et al., 2015; Qin et al., 2016; Wu et al., 2016; Kim et al., 2018; Ma et al., 2021).

**Figure 3:**
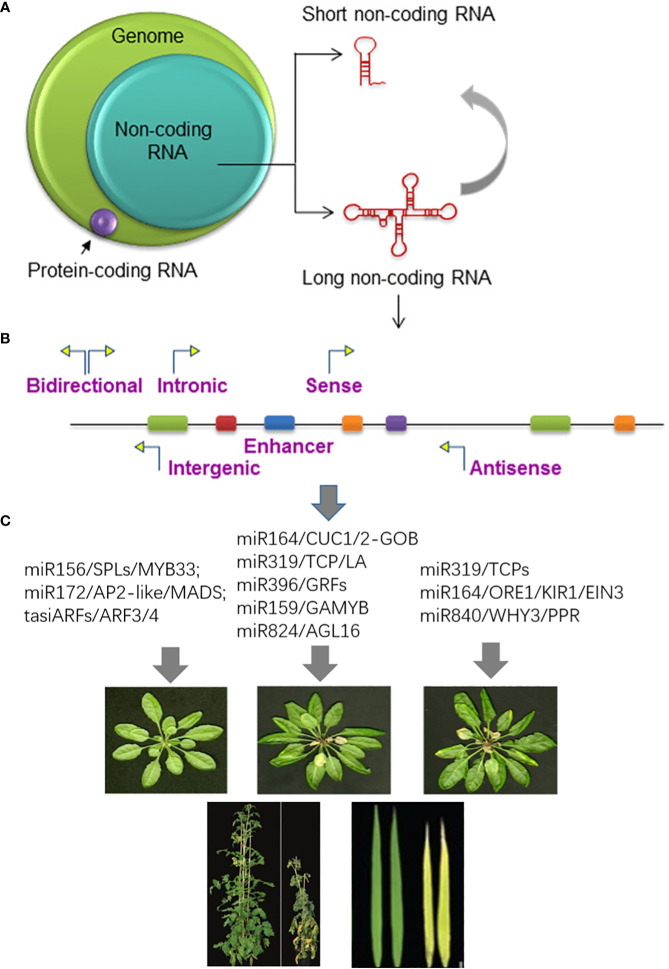
The role of microRNA in plant senescence regulation. **(A)** A high portion of the eukaryote genome is non-coding RNAs. Non-coding RNAs can be classified into small RNAs (18-30 nt) and long non-coding RNAs (lncRNAs); **(B)** Functions of non-coding RNA; **(C)** MicroRNAs and their regulatory circuits are involved in many aspects of plant senescence. Each panel represents plants at different developmental stages and demonstrates traits regulated by senescence-associated microRNAs. Before leaf senescence, overexpression of miR156 delays flowering and enhances vegetative growth through the targeting of transcription factors (TFs) SQUAMOSA PROMOTER BINDING PROTEIN-LIKE/SQUAMOSA PROMOTER BINDING PROTEIN (SPL/SBP). miR172 induces flowering by decreasing the expression of APETALA2 (AP2). In the shoot apical meristem (SAM), miR172 and SPL TFs work together to induce global proliferative arrest (GPA). At the onset of senescence, miR396 regulates leaf size and longevity by spatially and temporarily suppressing the expression of growth-regulating factor (GRF) TFs. miR164 specifically targets ORESARA1 (ORE1) and KIRA1 (KIR1), which promote flower senescence. ORE1 triggers age-dependent cell death and leaf de-greening, while KIR1 regulates stems cell death. miR319 plays a negative regulatory role in leaf senescence by targeting TEOSINTE BRANCHED/CYCLOIDEA/PCF (TCP) TFs. The TCPTFs, in turn, restrict vegetative growth by inhibiting cell divisions. By targeting both PPR and WHY3, miR840 cleaves the PPR transcript and inhibits WHY3 protein accumulation, thereby synergistically promoting leaf senescence.

Moreover, some miRNAs are associated with senescence induced by environmental stresses, such as flooding, darkness, extreme temperatures, and nutrition deficiencies. Under high light stress conditions, miR398 targets the CU/Zn superoxide dismutase, leading to leaf senescence (Hao et al., 2022). Authors reported that miR408 retarded dark-induced leaf senescence by repressing plastocyanin (PCY)- SAG14 module, which located on the endomembrane. However, phytochrome interacting factor 3/4/5 (PIF3/4/5) directly bound to the miR408 promoter and repressed its expression to regulate copper translocation to PCY-SAG14 during dark-induced leaf senescence. These findings suggest that the PCY-SAG14 module, which mediates intracellular copper homeostasis, plays an important regulatory role in dark-induced leaf senescence. Mishra et al. (2022) discovered a unique module, miR775-GALT9, during post-submergence recovery. They modulated the ethylene and ABA pathways to regulate the senescence of leaves in *Arabidopsis*. MIM775 transgenic lines exhibited accelerated senescence during post-submergence recovery, while miR775-overexpresing (Oe1) and *galt9* transgenic lines showed delayed senescence. The expression of *SAG29*, *SAG12*, and *ORE1* decreased in miR775-Oe1 and *galt9* transgenic lines, while their expression enhanced in MIM775-1 lines, suggesting that the miR775-GALT9 module regulated the expression of *SAGs* to control senescence during post-submergence recovery in *Arabidopsis*. In crop plants, very limited miRNA was reported their detail functions related to leaf senescence. A novel tomato miRNA known as SlymiR208 was identified. When it is overexpressed, the expression of *SlIPT2* and *SlIPT4* was significantly reduced, and endogenous CK concentrations in leaves was declined, leading to premature leaf senescence, which was in concurrence with the phenotype of *Isopentenyltransferases 4* (*IPT4*)-silenced lines, showing early leaf aging (Zhang et al., 2020). Another example in tomato, miR171b induced by exogenous melatonin directly bound to the α-glucan water dikinase (GWD) gene and activated it expression and increasingly catalyzed starch degradation and leaf senescence after prolonged carbon starvation. In addition, Wang et al. (2021) reported that MiR319 targeted *OsGAmyb* and *OsTCP21* to negatively regulate rice tillering and gain yield (Wang et al., 2021) (**Figure 3**).

### 6.2 LncRNAs is involved in stresses-induced senescence of plant

Long noncoding RNA (lncRNA) is a recently discovered class of epigenetic regulator involved in regulating gene expression during plant development and in response to stresses. Dynamic landscapes of long noncoding RNAs were observed in *Arabidopsis* leaves and rice flag leaves during aging (Huang et al., 2021; Kim et al., 2022). Above, a conserved miR164-NAC regulatory pathway was identified as being involved in leaf senescence. In this pathway, lncRNA MSTRG.62092.1 acts as a competing endogenous RNA (ceRNA) by binding to miR164a and miR164e to regulate three downstream transcription factors. Additionally, two other lncRNAs, MSTRG.31014.21 and MSTRG.31014.36, potentially regulate the abscisic acid biosynthetic gene *OsNCED4* (BGIOSGA025169) and the NAC family gene BGIOSGA016313 through osa-miR5809 (Huang et al., 2021). Further investigation was conducted on senescence-associated lncRNAs in *Medicago truncatula* nodules using high throughput strand specific RNA-seq. The analysis revealed that more than 60% of lncRNAs in the nodules were shown to associate with transposable elements, particularly the TIR/Mutator and Helitron DNA transposons families. It was predicted that 49 differentially expressed lncRNAs (DElncRNAs) were targeted by microRNAs. Notably, the majority of differently expressed target genes of DElncRNAs were related to the membrane component, with almost half of these genes involved in transporting organic material. These findings strongly suggest that DElncRNAs play a crucial role in substance transport across membranes during nodule senescence (Yu et al., 2022). Another example, LncRNA ASCO, a homology of GmENOD40 in *Arabidopsis*, competitively interacted with the RNA-binding protein NSRs in the nucleus in response to growth hormone (IAA) signals, thereby regulating alternative splicing of NSRs-targeted pre-mRNA to influence root development and senescence (Bardou et al., 2014). In addition, Pathogen effector can induce the transcription of an lncRNA named ELENA1 in *Arabidopsis* cells, which ELENA1 accumulates MED19a protein in the promoter region of the downstream gene *PR1* through interaction, enhancing pathogen induced cell death in the innate immunity of plants (Seo et al., 2017). Up to now, the action aspect of lncRNA in leaf senescence is still limited.

## 7 RNA methylation and plant senescence

RNA modification, such as N6-methyladenosine (m6A) and 5-methylcytosine (m5C), are another kind of epigenetic regulation. With the advances in technology of mass spectrometry, next-generation sequencing, and genome mapping, detection of mRNA modifications has become more precise. Up to now, more than 170 various RNA modifications were reported. The increasing reports are mostly focused on chemical modifications of specific mRNA nucleotides like m6A and m5C, which can enhance or reduce the binding activity of the regulatory factors, such as transcription factors, RNA binding proteins, and non-coding RNAs. Here, we try to summarize the chemical mRNA modifications, specifically m6A affect plant senescence (Gabriel et al., 2019; Kumar and Mohapatra, 2021; Shen et al., 2023).

N6-methyladenosine (m6A) is the most widespread, plentiful and conserved internal cotranscriptional modification in eukaryotic RNAs, especially within higher eukaryotic cells. m6A modification is modified by the m6A methyltransferases (writer) and removed by the demethylases (erasers). It is recognized by m6A-binding proteins (readers) (Jiang et al., 2021). In plants, m6A was one of best characterized mRNA modifications. m6A has an evolutionarily conserved m6A methyltransferase complex, which is necessary for m6A deposition to target transcripts. In *Arabidopsis*, this complex contains two core methyltransferases and several accessory proteins. The former includes mRNA adenosine methylase MTA and mRNA adenosine methylase MTB, which are orthologue of METTL3 and METTL14, respectively.The latter includes FKBP12 INTERACTING PROTEIN 37KD (FIP37) and HAKAI. m6A methyltransferase deposits m6A on the region closed to stop codons of mRNAs and the region with a RRACH sequence in 3′UTRs, respectively, like in mammalian, responding for most of m6A enrichment of mRNA. Another m6A methyltransferase FIO1 that is orthologue of METTL16, can add m6A modifications on the regions with a YHAGA (Y = C/U) motif of coding sequence transcript around stop codons or with a RRACH in 3’UTRs, affecting all m6A levels in plant (Xu et al., 2021; Wang et al., 2022), which is inconsistence with METLL16 function on a UACm6AGA GAA sequence existed in a stem-loop structure in mammalian (Pendleton et al., 2017; Warda et al., 2017). Nevertheless, both METTL16 in mammalian and FIO1 in plant can enrich m6A to the small noncoding nuclear RNA U6 of spliceosome.

Recent research suggests that the specificity of m6A with its target could be developed through interaction of m6A writers and other writer-associated proteins such as RNA binding protein (RBP), named m6A recruiters with specific transcripts in plants. For examples, it has reported that the RBP FCA interacted with MTA, MTB, and FIP37 in the complex and acted m6A enrichment on the antisense transcript COOLAIR of noncoding RNA during *Arabidopsis* flowering (Xu et al., 2021). The m6A writer OsFIP37can be recruited by RBP OsFIP37-assocated protein 1 (OsFAP1) to add m6A on OsYUCCA3 transcripts during rice male meiosis (Cheng et al., 2022). Moreover, other m6A writers MTA, MTB, and FIP37 can be recruited by a characterized m6A recruiter cryptochrome 2 (CRY2), to install m6A on the transcripts of central circadian clock oscillator genes (Wang et al., 2021). Although these studies showed many members of m6A-depositing machineries performed transcript-specific m6A methylation, they did not directly connect with cell senescence of plant, in fact, m6A recruiter cryptochrome 2 (CRY2) has been reported that it represses leaf senescence in response to blue light (Kozuka et al., 2023). In dark-induced leaf senescence in barley, the gene expression microarrays of barley plants exposed to dark induced senescence (Sobieszczuk-Nowicka et al., 2018) shows six RNA methyltransferases from the group of the selected genes with their isoforms, specifically upregulated in reaction to dark-induced senescence, but not developmental senescence. The identified upregulated genes include RNA methyltransferases of different RNA types, namely, enzymes modifying mRNA, tRNA, and rRNA. Authors suggested that discoveries of m6A RNA modification changes in certain RNA species in different stages of leaf senescence may uncover the role of such modifications in metabolic reprogramming (Rudy et al., 2023).

The integrative analyses of m6A methylomes and transcriptome at different development stage in various tissues in *Arabidopsis*, including roots, rosette leaves, and flowers showed that the fractions of transcripts apparently enriched m6A modifications in senescent tissues, and m6A enriched level is much higher than transcript altered level, suggesting that m6A may be more important regulator in organ differentiation and programed cell death process (Wan et al., 2015). Furthermore, m6A modifications are affected by various (a)biotic-stresses, such as heat, salt, and drought induced cell senescence (Liu et al., 2020; Hu et al., 2021; Hou et al., 2022), as well as biotic stresses such as virus and fungal diseases induced senescence (Zhang et al., 2021; Guo et al., 2022). These stresses significantly induce m6A redistribution on selected transcripts (Shen et al., 2023), but its distribution pattern in the 3′UTR and around stop codons did not be altered, supposing that dynamics of m6A in various situations might be partially resulted from unbalance of levels of m6A writers and erasers. It supposes that specific m6A RNA binding proteins may function at distinct tissue-or organ- developmental stages or under different environmental stimuli to guide m6A writers to various sets of transcripts, generating development- or stimulus- dependent m6A methylomes (Shen et al., 2023).

**Figure 4:**
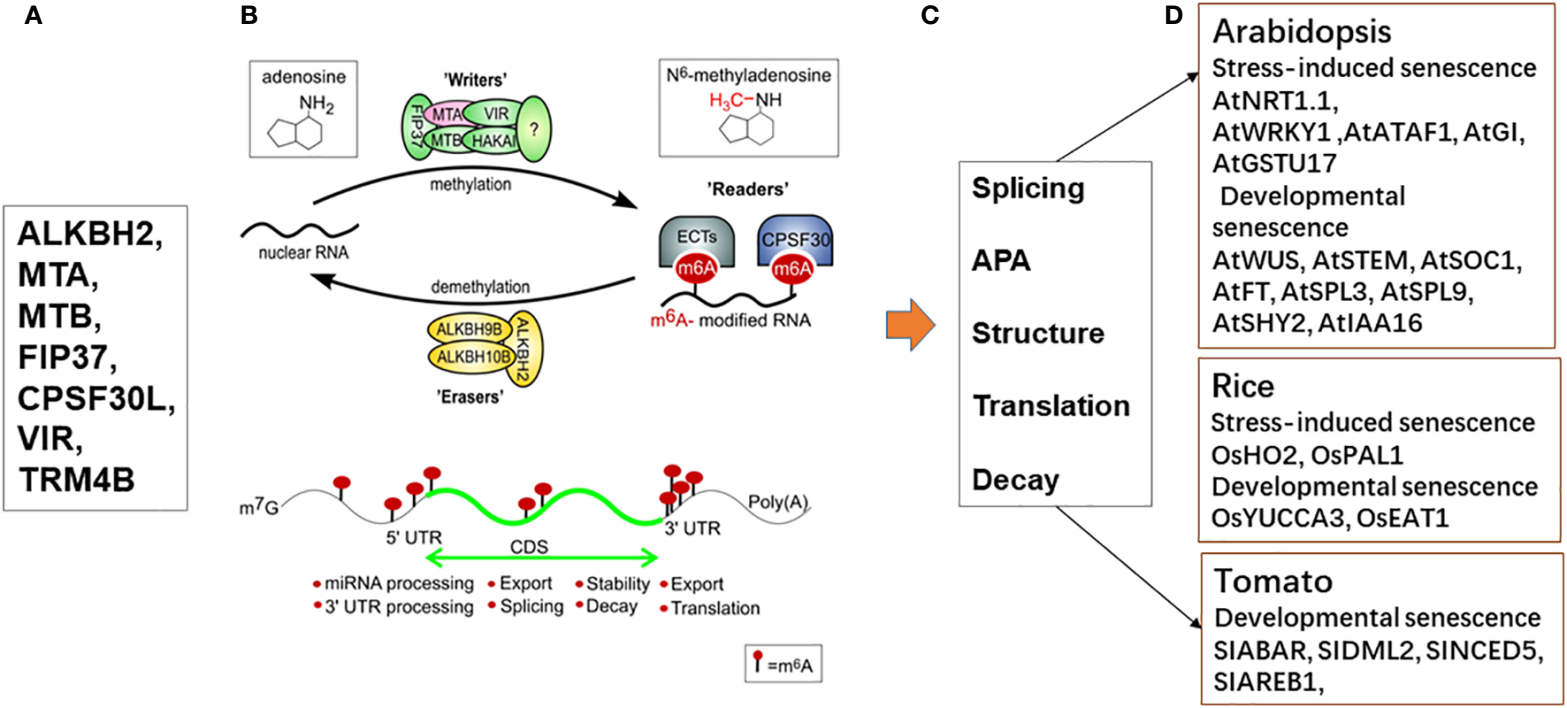
Regulation of plant’s development and response to stresses in various plant species by RNA modifications (m6A). **(A)** The RNA modifications (m6A) were regulated by writers (MTA, MTB, FIP37, VIRILIZER, and HAKAI), erasers (ALKBH2, ALKBH9B, and ALKBH10B) and reader proteins (ECT2/3/4 and CPSF30); **(B)** Regulation of mRNA m6A modification in plants through the action of a network of m6A writers (methyltransferase), erasers (demethylase), and reader proteins. The m6A writer complex consists of the proteins MTA, MTB, FIP37, VIRILIZER, and HAKAI. The m6A modifications can be added by writers and removed by erasers within the nucleus. The m6A readers bound specifically to m6A sites and mediate distinct functions. The expression of subunit MTA (red) was shown to be upregulated during dark induced leaf senescence, previously (Sobieszczuk-Nowicka et al., 2018). **(C)** Typical m6A distribution in regions of an mRNA and its readout affects mRNA fates, including splicing, APA, structure, translation, decay (Zheng et al., 2020; Sokpor Rudy et al., 2023). **(D)** Several candidate genes in developmental senescence and stress induced senescence of various plant species were affected by RNA modification.

In addition, RNA modifications control plant mRNA fate through affecting mRNA metabolism, including alternative splicing (AS), alternative polyadenylation (APA), protein folding, translation, localization, RNA transport, and RNA decay (Gabriel et al., 2019; Kumar and Mohapatra, 2021; Shen et al., 2023). We summarized most of m6A related functions from different plant species such as *Arabidopsis*, rice, and tomato of the RNA modification effectors deficient-mutants (Shen et al., 2023) (**Figure 4**). It indicates that the effects of RNA modification on mRNA metabolism finally affect widely physiological processes during plant development and in response to stresses including developmental senescence and stresses-induced senescence. Although above studies have revealed dynamic redistribution m6A methylations in tissue-, age-, and stress-dependent manners in plants, and SAGs mRNA fate was determined by RNA modification mediating RNA metabolism, the underlying mechanisms so far remain elusive.

## 8 Epigenetically manipulation and application of plant senescence

Summarily, DNA methylation enrichment accompanies with a delaying leaf senescence, histone acetylation enrichment increases with leaf senescence, and chromatin remodeling and related factors affect stress-induced senescence. Using large-scale *Arabidopsis* expression data reported by Breeze et al. (Breeze et al., 2011) and available *Arabidopsis* and rice leaf senescence-related epigenetomic data (Brusslan et al., 2015; Li et al., 2016; Zhang et al., 2022), we clarified the transcription pattern of SAGs, which are known to be involved in plant DNA methylation, histone modification, non-coding RNA regulation processes. Genes such as *AGO10* (one of components of the RNA-induced silencing complex), *MET1* and *ROS1* were significantly regulated at the transcription level in dark-induced leaf senescence (Ostrowska-Mazurek et al., 2020); HAD6, HDA9, HDA15, several of histone deacetylases, regulated leaf senescence and flowering (Huang et al., 2022), histone acetyltransferase HAC1 is highly expressed in both dark-induced leaf senescence and developmental leaf senescence (Brusslan et al., 2012); REF6, one of histone demethylase, with BRM to regulate leaf senescence (Li et al., 2016) (**Table 1**). This suggests the opportunity of an additional switch between cell survival and cell death. Thus, understanding the mechanism of epigenetic modifications during plant aging and response to stresses is highly significant for cultivating anti-senescent vegetable varieties, improving crop biomass and product quality in raising agricultural production.

**Table 1:** Epigenetic regulators of leaf senescence in Arabidopsis, rice and tomato.

Type	Gene Name	Functional Category	Mutant Phenotype	Regulation of senescence	References
DNA methylation	*MET1*	Arabidopsis DNA Methyltransferase	global DNA hypomethylation; delayed senescence	Positive	Kim et al., 2008
	*DMR1/2;CMT3*	Arabidopsis DNA Methyltransferase	hypomethylated triple mutant *dmr1/2 cmt3* (*ddc*) faster leaf senescence ; enhanced nitrogen remobilization	Negative	Vatov et al., 2022
	*OsDRM2*	Rice DNA Methyltransferase	delayed or no heading; other growth defects;near-complete CHH methylation loss;derepression of small transposable elements	Positive	Moritoh et al., 2012; Tan et al., 2016
	*SlDRM7*	Tomato DNA Methyltransferase	pleiotropic developmental defects;small and interveinal chlorosis leaves	Negative	Wen et al., 2022
DNA demethylation	*DML3*	Arabidopsis DNA Demethylase	CG hypermethylation at the promoters of SAGs; delayed leaf senescence	Positive	Yuan et al., 2020
	*ROS1*	Arabidopsis DNA Demethylase	hypermethylated mutant *ros1* faster leaf senescence ; enhanced nitrogen remobilization	Negative	Vatov et al., 2022
Histone methylation	*SUVH2*	Arabidopsis histone methyltransferase	overexpression of SUVH2 delayed leaf senescence	Negative	Ay et al., 2009
Histone demethylation	*JMJ16*	Arabidopsis H3K4 demethylase	*jmj16* mutant increased H3K4me3 at WRKY53 and SAG201; early leaf senescence	Negative	Liu et al., 2019
	*REF6*	Arabidopsis H3K27me3 demethylase	Delayed leaf senescence; H3K27me3 hypermethylation	Positive	Wang et al., 2019
	*SlJMJ4*	Tomato H3K27 demethylase	premature leaf senescence; enhanced dark-and ABA- induced leaf senescence	Negative	Ding et al., 2022
Histone acetylation	*HAC1*	Arabidopsis histone acetyltransferase	promoting leaf senescence; regulating the expression of ERF022 by H3K9ac enrichment	Positive	Hinckley et al., 2019
Histone deacetylation	*AtHD1*	Arabidopsis histone deacetylase	reduced *AtHD1* transcript level; increased H4ac; early leaf senescence	Negative	Tian and Chen, 2001
	*HDA6*	Arabidopsis histone deacetylase	higher levels of H3ac; prolonged leaf lifespan	Positive	Wu et al., 2008
	*HDA9*	Arabidopsis histone deacetylase	delayed leaf senescence; increased H3K27ac levels	Positive	Chen et al., 2016
	*HDA15*	Arabidopsis histone deacetylase	delayed leaf senescence; moving out H3K9ac at LOX2, WRKY53, ELF4	Negative	Huang et al., 2018; Huang et al., 2022
	*OsHDA710*	Rice histone deacetylase	overexpression of OsHDA710 delays leaf senescence	Negative	Zhao et al., 2020
	*OsSRT1*	Rice H3K9 deacetylase	OsSRT1 RNAi line accelerated leaf senescence; increased H3K9ac at *OsSAG12; deacetylation of H3K9ac in OsPME1*	Negative	Huang et al. 2007; Zhong et al., 2013
Chromatin remodeling	*ORE7/ESC*	chromatin architecture-controlling protein	overexpression of ORE7 *SAGs* inhibition; delayed senescence.	Negative	Lim et al., 2007
	*DRD1*	chromatin remodeling protein 1	overexpression of DRD1 *SAGs* inhibition; delayed senescence	Negative	Cho et al., 2016
	*DDM1*	ATP-dependent DNA helicase DDM1	SAGs inhibition; delayed senescence.	Negative	Cho et al., 2016
	*BRM*	catalytic ATPase subunits of the SWI2/SNF2 complex	delayed leaf senescence	Negative	Archacki et al., 2013; Li et al., 2016
non-conding RNA	miR319	miRNA	targetsTCP TFs;regulate WRKY53	Negative	Schommer et al., 2008
	miR164	miRNA	prevent premature overexpression of ORE1	Negative	Kim et al., 2009; Li et al., 2013
	miR840	miRNA	overexpression of MIR840 promotes leaf senescence;cleaves the PPR transcript; inhibits WHY3 protein accumulation	Positive	Ren et al., 2022
	miR408	miRNA	retards dark-induced leaf senescence; repressing plastocyanin (PCY)- SAG14	Negative	Hao et al., 2022
	miR775	miRNA	overexpression of miR775 delayed leaf senescence; miR775-GALT9 module regulate SAGs	Negative	Mishra et al., 2022
	SlymiR208	miRNA	overexpression of SlymiR208 premature leaf senescence;reduced the expression of SlIPT2 and SlIPT4	Positive	Zhang et al., 2020
	SlymiR171b	miRNA	overexpression of miR171b;ameliorates carbon starvation‐induced leaf chlorosis;inhibited the expression of GWD	Negative	Wang et al., 2021

From all described above of multiple roles of epigenetic modification and epitranscriptome in plant developmental senescence and environmental stress-induced senescence adaptations in diverse plant species. Plant molecular genetic manipulation can operate both genetic variation and epi-allelic variation as sources of agricultural trait variations. Manipulating the epigenetome and its factors is a hopeful breeding strategy for crop improvement. A typical example is that expressing the human m6A demethylase, a human enzyme mediating RNA m6 A demethylation and originally identified as a fat mass- and obesity-associated protein FTO, in rice and potato cells, in greenhouse conditions, rice with transgenic FTO expression increased grain production by nearly 300%. Transgenic expression of FTO in rice and potato during field tests increased yield and biomass by about 50%. FTO has no effect on mature cell size, cell proliferation, shoot meristem, root diameter, plant height, or ploidy. However, it does stimulate the production of tiller buds and root meristem cell proliferation, as well as photosynthetic performance and tolerance to drought (Yu et al., 2021). Epigenetic variants controlling potential agronomic traits have been shown in several crop species, for examples, sex determination in melon (Martin et al., 2009), anthocyanin production in apple (Telias et al., 2011), increased seed protein/oil ratio in oilseed rape (Long et al., 2011), a dwarf phenotype in rice (Chen and Zhou, 2013), and fruit ripening in tomato (Liu et al., 2015), as well as an early flowering in strawberry and an early leaf senescence in Pak Choi after treatment with inhibitor of DNA methylation (Xu et al., 2016; Li et al., 2022), and drought and salt tolerance in rice (Garg et al., 2015). Tomato, *Solanum lycopersicum*, is a fleshy, climacteric fruit model plant, as well as an economically important crop plant. Indeed, knocking down the expression of *SlORE1S02*, an AtORE1 orthologous with a disrupted miR164 hybridization site, led to delayed senescence, which was evidenced by a stay-green phenotype (Lira et al., 2017).

Besides, in recent years, the developed DNA/RNA editing techniques have promoted the expanding of multiple strategies for epigenetic and epitranscriptome editing of crops at different level (Zheng et al., 2020; Shen and Yu, 2021; Shen et al., 2023). It can be summarized as: (1), with developing of CRISPR/Cas9-mediated gene editing technique, the activity of enzymes or factors related to DNA-modification, histone-modification and RNA-modification can be controlled, thus generating new traits mediated by DNA, histone, RNA modifications become possible. (2), DNA and RNA modification sites of specific targets could be directly edited through accurate base editors, for example, the adenine base editor contains the catalytically inactive CRISPR/Cas9 protein and an engineered adenosine deaminase causing A to G substitution (Kang et al., 2018; Kim, 2018). (3), By using the catalytically inactivated Cas13, DNA or RNA modifications could be specifically generated or removed on specific target sites similar to mammalian cells (Wilson et al., 2020), for instance, when dCas13 is fused to m6A demethylases ALKBH5, the light-sensitive protein CBIN and its adaptor CRY2 could make targeted RNA demethylation in plants (Li et al., 2020; Zhao et al., 2020). (4), By using genetic manipulation of histone modification writers (methyltransferase and acethyltransferase) and esters (methylase and acethylase), histone modification mark and chromatin remodeling status could also be created or remove to control the expression of specific target gene (e.g. SAGs) related to agronomic characters in crops.

## 9 Conclusions and prospect

Over the last decade, there have been significantly progressed in the mechanistic understanding of epigenetic modifications and epitranscriptome, including dynamics of H3K4me3 and H3K9ac, dynamics of non-coding RNAs, m6A dynamics during tissue and organ development and under stresses in the model plant *Arabidopsis* and rice, as well as other plant species. Current evidences strongly suggest that epigenetic marks play an essential role of post-transcriptional gene regulation that determines RNA destiny and finally influence plant developmental senescence and adaptation to different environmental stresses-induced senescence. However, our understanding of action mechanism of plant epigenetome and epitranscriptome in cell senescence field are still very limited. Many challenging questions regarding the SAG targets selected and functional aspect of epigenetomic and epitranscriptomic marks remain to be answered. How many methyltransferases or demethylases, acethyltransferlases or acetylases (writers or erasers) are specifically modified SAGs? How do these writers and erasers determine their targets in distinct physiological procedure and various tissues and organs, at different developmental stage, or under multiple stresses? How do reader proteins recognize their targets (SAGs) and carry out their roles in following RNA metabolic processes? At the same time, when we are applying epigenetomic and epitranscriptome editing in crop biotechnology, numerous challenges must be faced. First, it is required to develop a novel techniques to analyze DNA modification, histone modification and RNA modification dynamics from the tissue levels to the cellular levels even at single-nucleotide resolution or at single-amino acid resolution. For example, DNA/RNA epigenetic modified sites at single-base resolution are largely unknown in crops, the techniques of TCTA-seq, miCLIP, MAZTER-seq, m6A- SAC-seq, and Nanopore DRS are necessary to be developed to accurately detect DNA/RNA methylation and interrogate modified sites at single-nucleotide resolution in crops. The biological actions of erasing/writing epigenomic marks at specific SAGs, histone epigenetic mark targeted to a specific SAGs locus are mostly unidentified in plants, especially, epitranscriptome mark at SAGs transcript is blank yet. Although many epigenetic enzymes and factors of DNA or RNA modifications or histone modification have been reported in several crops (Wu et al., 2022), their integrative action mechanisms in controlling senescence related gene expression remains largely elusive. In DNA/RNA-editing systems, a concern with transgenerational stability of epigenetic variation did not be solved, the specificity of their targets and the efficiency of DNA/RNA editing have yet to be established and examined in most crops.

## Author contributions

YZ: Writing – original draft. DH: Writing – original draft. YM: Writing – original draft, Writing – review and editing.
